# Association between childhood anthropometric indicators and bone mineral density in adulthood

**DOI:** 10.1590/1984-0462/2024/42/2023026

**Published:** 2023-10-23

**Authors:** Mileny Caroline Menezes de Freitas, Julio Cesar da Costa, Cynthia Correa Lopes Barbosa, Lidyane Ferreira Zambrin, Catiana Leila Possamai Romanzini, Marcelo Romanzini, Enio Ricardo Vaz Ronque

**Affiliations:** a Universidade Estadual de Londrina, Londrina, PR, Brazil.; b Universidade Tecnológica Federal do Paraná, Apucarana, PR, Brazil; c Universidade Federal de Mato Grosso do Sul, Corumbá, MS, Brazil.

**Keywords:** Bone density, Body mass index, Body weight, Child, Adult, Densidade óssea, Índice de massa corporal, Peso corporal, Crianças, Adulto

## Abstract

**Objective:**

This study aimed to verify the association between childhood anthropometric indicators and areal bone mineral density (aBMD) in adulthood.

**Methods:**

Repeated measures of 137 subjects (68 females) were obtained in childhood (9.2±1.5 years of age) and adulthood (22.3±1.7 years of age). aBMD (g/cm^2^) was assessed for whole body, lumbar spine, upper and lower limbs, and femoral neck in adulthood using dual-energy X-ray absorptiometry. Anthropometric measurements of body weight (BW), height, triceps and subscapular skinfolds were obtained in childhood. The anthropometric indicators used were BW, body mass index (BMI), and sum of skinfolds (ΣSF). Simple linear regression was used to assess the association between childhood anthropometric indicators and aBMD in adulthood, controlled by chronological age and stratified by sex, with 5% statistical significance.

**Results:**

In females, multiple associations were observed between anthropometric indicators and aBMD, with higher coefficients for BMI (β=0.020; R^2^=0.20; p<0.01 for right femoral neck to β=0.008; R^2^=0.16; p<0.01 for upper limbs), followed by BW (β=0.003; R^2^=0.21; p<0.01 for upper limbs to β=0.008; R^2^=0.20; p<0.01 for right femoral neck) and ΣSF (β=0.001; R^2^=0.06; p<0.01 for upper limbs to β=0.005; R^2^=0.12; p<0.01 for right femoral neck). In males, associations were observed only for the lumbar spine region (β=0.016; R^2^=0.09 for BMI to β=0.004; R^2^=0.06; p<0.01 for ΣSF).

**Conclusions:**

Anthropometric indicators of childhood proved to be sensitive predictors of aBMD in adulthood, especially in females. BMI indicated a greater association with aBMD in both sexes.

## RESUMO

**Objetivo:**

O objetivo do estudo foi verificar a associação entre os indicadores antropométricos da infância com a área da densidade mineral óssea (aDMO) na idade adulta.

**Métodos:**

Medidas repetidas de 137 sujeitos (68 do sexo feminino) foram obtidos na infância (9,2±1,5 anos de idade) e idade adulta (22,3±1,7 anos de idade). A aDMO (g/cm^2^) foi avaliada para todo o corpo, coluna lombar, membros superiores e inferiores e colo do fêmur na idade adulta usando a absorciometria radiológica de dupla energia (DXA). Medidas antropométricas de peso corporal, estatura e dobras cutâneas das regiões tricipital e subescapular foram obtidas na infância. Os indicadores antropométricos utilizados para as análises foram o peso corporal (PC), o índice de massa corporal (IMC) e o somatório de dobras cutâneas (ΣDC). Regressão linear simples controlada pela idade e estratificada por sexo foi empregada para avaliar a associação entre os indicadores antropométricos do período da infância na aDMO na idade adulta, com significância estatística de 5%.

**Resultados:**

No sexo feminino, múltiplas associações foram observadas entre os indicadores antropométricos e a aDMO, com maiores coeficientes para IMC (β=0,020; R^2^=0,20; p<0.01 para colo do fêmur direito a β=0,008; R^2^=0,16; p<0,01 para membros superiores), seguido da PC (β=0,003; r^2^=0,21; p<0,01 para membros superiores a β=0,008; r^2^=0,20; p<0,01 para colo do fêmur direito) e ΣDC (β=0,001; R^2^=0,06; p<0,01 para membros superiores a β=0,005; R^2^=0,12; p<0,01 para colo do fêmur direito). No sexo masculino, associações ocorreram apenas na região da coluna (β=0,016; R^2^=0,09 para IMC a β=0,004; R^2^=0,06; p<0,01 para ΣDC).

**Conclusões:**

Indicadores antropométricos da infância mostraram ser sensíveis preditores da aDMO na idade adulta, especialmente no sexo feminino. O IMC indicou maior associação com a aDMO em ambos os sexos.

## INTRODUCTION

Peak bone mass (BM) is characterized by the maximum amount of bone tissue reached when geometric properties cease to change, and at around 30 years of age, it reaches a plateau.^1–3^ The BM accumulation determined in this period reduces the risk of fractures and also has the potential to delay the development of osteoporosis at more advanced ages.^1^

Childhood and adolescence are the periods of life marked by a rapid increase in the speed of BM gains, specifically in adolescence during peak height velocity, which is essential for optimizing BM gains.^2,3^ The interaction between aspects such as body weight (BW), the different tissues that compose it, and bone metabolism is complex and multifactorial. Thus, the effect of several morphological factors on BM has been mediated by mechanical and biochemical aspects.^4^

The interaction between BW and BM is based on the mechanostatic theory, that is, the bone undergoes internal deformations and mechanical adaptations according to the load to which it is subjected,^5^ and on the mechanotransduction theory, in which osteocytes transmit mechanical stimuli that in turn recruit osteoblasts and osteoclasts that modulate BM and structure.^6^ Thus, during daily activities, the mechanical load imposed by BW on bone tissues seems to be associated with the amount of accumulated BM.^7,8^

Considering the above, previous cross-sectional studies have shown that in childhood and adolescence, both BMI and BW are positively associated with areal bone, bone mineral density (BMD), and bone mineral content (BMC).^9,10^ Additionally, longitudinal studies have shown that BMI in early adolescence is positively associated with BMD at the end of this period,^11,12^ as well as in the transition to adulthood.^13^ Regarding late adolescence, BMI also seems to be positively associated with BMD in adulthood.^14^ Although these results reveal a contribution of BMI to BM, there is a scarcity of investigations on the impact of BMI and BW in childhood and BM in adulthood.

Few studies that have investigated this topic have shown partially divergent results, indicating positive associations between BMI in childhood and BM in adulthood in both sexes^15,16^ or only in males.^17^ In addition, in obese children and adolescents, visceral fat is inversely associated with BMD,^18^ while body adiposity estimated by subscapular skinfolds is positively associated with BMD in the lumbar spine and hip regions.^19^ Another important aspect is the lack of investigation of the association of these anthropometric indicators with different bone regions, considering that mechanical loads can generate different adaptations in the BMD of athletes^20^ and of older adults^21^ and that studies involving young people have been mainly focused on specific regions of the lumbar spine and femoral neck.^12,15,16,19^

In this context, it is important to investigate the possible impact of childhood anthropometric indicators on bone health indicators in adults, considering that childhood is a potential phase for the development of these factors, and that this relationship also needs to be further elucidated.^22^ Thus, the present study aimed to verify the association between anthropometric indicators (BW, BMI, and sum of skinfolds [ΣSF]) in childhood with the areal bone mineral density (aBMD) in adulthood.

## METHOD

Data were extracted from the longitudinal study entitled “Physical fitness and sport participation in childhood and adolescence and biological and behavioral risk factors in adults: a 15-year longitudinal study.” Baseline was a mixed longitudinal study carried out in 2002, where students of both sexes aged 7–10 years from four different years of birth were selected (1992, 1993, 1994, and 1995), being followed annually from 2003 to 2006, with four age overlaps, as described in the previous study.^23^

Inclusion criteria, eligibility, and final sample definition for the follow-up are described in a previous study.^24^ Sampling included data from 142 adults evaluated in 2016. All participants, after being duly informed about the study objectives and the procedures to which they would be submitted, signed the informed consent form. This study was approved by the Research Ethics Committee of the State University of Londrina in accordance with the norms of Resolution 466/2012 of the National Health Council for research involving human beings (No. 1.340.735).

BW and height were measured according to procedures described by Gordon et al.^25^ At baseline, BW was measured on a Filizola digital platform scale, model ID-1500, and height was measured using a wooden stadiometer. During follow-up, these measurements were obtained using a digital platform scale, brand Seca, and a portable stadiometer, brand Harpenden. BMI was determined by the BW/stature ratio.^2^ In both phases, body adiposity was determined by the sum of skinfold measurements (ΣSF) of the triceps and subscapular regions, using a scientific Lange adipometer (Cambridge Scientific Industries Inc.), according to standards described by Harrison et al.^26^

aBMD was estimated using the dual emission x-ray absorptiometry (DXA) technique by a single certified technician, with Lunar DPX-MD+ equipment (GE Lunar Corporation, 726 Heartland Trail, Madison, WI 53717-1915, USA). Data were obtained using the software recommended by the manufacturer (enCORE version 4.00.145). For exams, individuals were instructed about contraindications, procedures, and appropriate clothing. A whole body scan was performed with participants in the supine position and aligned for approximately 15 to 20 min, and aBMD of the whole body, upper and lower limbs, spine, and right and left proximal femoral neck was estimated.

Data were presented as mean and standard deviation for sample characterization. Comparisons between sexes were performed using Student's t-test for independent samples. The Pearson correlation coefficient was used to analyze the association between BMI, BW, andΣ SF in childhood and aBMD in adults, and simple linear regression was used with chronological age control to verify the magnitude. Statistical procedures were performed using the SPSS software (IBM SPSS Statistics for Windows, 28.0, IBM Corp., Armonk, NY), and the statistical significance level adopted in analyses was 5%.

## RESULTS

The descriptive sample characteristics in childhood and adulthood are shown in Table 1. In childhood, there were no differences between sexes for anthropometric variables (p>0.05). In adulthood, all descriptive variables differed between boys and girls, except for age (p=0.58). aBMD indicators of the whole body, lumbar spine, upper limbs, lower limbs, and right and left femoral neck were higher in males when compared to females (p<0.001).

**Table 1:** Descriptive statistics and comparisons between males (n=69) and females (n=68) at childhood and adulthood.

		Childhood			Adulthood		
		Females	Males	p-value	Females	Males	p-value
Descriptive variables							
	Chronological age (years)	9.0±1.6	9.3±1.3	0.25	22.2±1.7	22.4±1.7	0.58
	Body weight (kg)	32.1±9.5	33.6±8.2	0.29	60.3±10.7	76.1±10.6	<0.001
	Height (cm)	134.8±11.6	136.0±9.3	0.50	164.6±6.7	176.5±6.0	<0.001
	Body mass index (kg/m^2)^	17.3±2.8	18.0±2.6	0.15	22.2±3.4	24.4±2.9	<0.001
	Σ Skinfolds (mm)	24.2±10.1	22.3±10.2	0.27	33.7±8.5	24.3±9.4	<0.001
aBMD							
	Whole body (g/cm^2^)	–	–		1.167±0.074	1.269±0.091	<0.001
	Lumbar spine (g/cm^2^)	–	–		1.127±0.115	1.168±0.119	0.021
	Upper limbs (g/cm^2^)	–	–		0.795±0.049	0.945±0.092	<0.001
	Lower limbs (g/cm^2^)	–	–		1.203±0.095	1.430±0.122	<0.001
	Right femoral neck (g/cm^2^)	–	–		1.038±0.124	1.165±0.184	<0.001
	Left femoral neck (g/cm^2^)	–	–		1.025±0.111	1.178±0.151	<0.001

aBMD: areal bone mineral density; Σskinfolds: sum of skinfolds; Student's t-test.

Childhood anthropometric indicators showed positive and low-magnitude correlations with aBMD in females in all investigated body anatomical regions, with variations from r=0.28 (p<0.05) for BW and right femoral neck to r=0.45 (p<0.001) for BMI and whole body. In males, positive and low-magnitude correlations were observed only in spine aBMD, between r=0.30 (p<0.05) and r=0.35 (p<0.001) for skinfolds and BW, respectively (Table 2).

**Table 2:** Correlation (95%CI) of childhood anthropometric indicators and adulthood aBMD.

Childhood Anthropometric indicators	Adulthood aBMD (g/cm^2^)					
Females						
	Whole body	Lumbar spine	Upper limbs	Lower limbs	Right femoral neck	Left femoral neck
BMI (kg/m^2^)	0.45*	0.39*	0.44*	0.45*	0.41*	0.42*
	(-0.24–0.62)	(0.11–0.53)	(0.22–0.61)	(0.24–0.62)	(0.18–0.59)	(0.20–0.60)
BW (kg)	0.38*	0.31*	0.43*	0.41*	0.29†	0.31†
	(0.16–0.57)	(0.08–0.51)	(0.22–0.61)	(0.19–0.59)	(0.06–0.50)	(0.08–0.51)
ΣSF (mm)	0.37*	0.31†	0.30†	0.37*	0.35*	0.37*
	(0.15–0.56)	(0.07–0.51)	(0.07–0.51)	(0.14–0.56)	(0.13–0.55)	(0.14–0.56)

Males						
	Whole body	Lumbar spine	Upper limbs	Lower limbs	Right femoral neck	Left femoral neck
BMI (kg/m^2^)	0.13	0.34*	0.08	-0.04	-0.09	-0.09
	(-0.11–0.35)	(0.11–0.53)	(-0.16–0.31)	(-0.28–0.19)	(-0.32–0.15)	(-0.33–0.14)
BW (kg)	0.18	0.36†	0.16	0.04	0.01	-0.13
	(-0.06–0.40)	(0.13–0.55)	(-0.08–0.38)	(-0.20–0.28)	(-0.23–0.25)	(-0.36–0.09)
ΣSF (mm)	0.09	0.30†	0.06	-0.07	-0.09	-0.14
	(-0.15–0.43)	(0.06–0.50)	(-0.18–0.29)	(-0.30–0.17)	(-0.32–0.15)	(-0.37–0.09)

aBMD: areal bone mineral density; BW: body weight; ΣSF: sum of skinfolds; BMI: body mass index. Pearson's correlation and confidence intervals

* (p<0.01;

† p<0.05).

**Figure 1:**
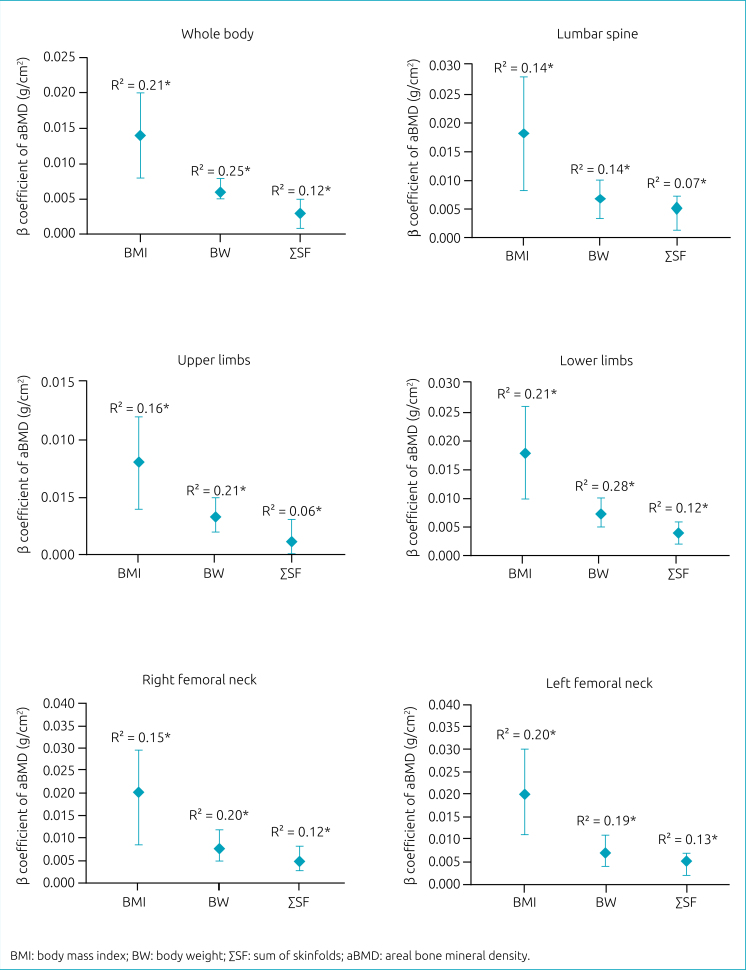
Association between body mass index, body weight, and sum of skinfolds at childhood and areal bone mineral density at adulthood in females (n=68). Simple linear regression (p<0.01); *adjusted for chronological age.

Figure 1 illustrates associations between BMI, BW, and ΣSF in childhood and aBMD in adulthood for females. It is noteworthy that associations were positive, with higher beta coefficients for BMI, followed by BW and ΣSF, with BMI explaining between 14 and 21% (p<0.01), BW between 14 and 28% (p<0.01), and ΣSF between 6 and 12% (p<0.01) of aBMD variation in several anatomical regions in adulthood. Although the three childhood anthropometric indicators were shown to be predictors of aBMD in adulthood, BMI was the factor that most contributed to aBMD, while ΣSF was the factor with the lowest contribution.

**Figure 2:**
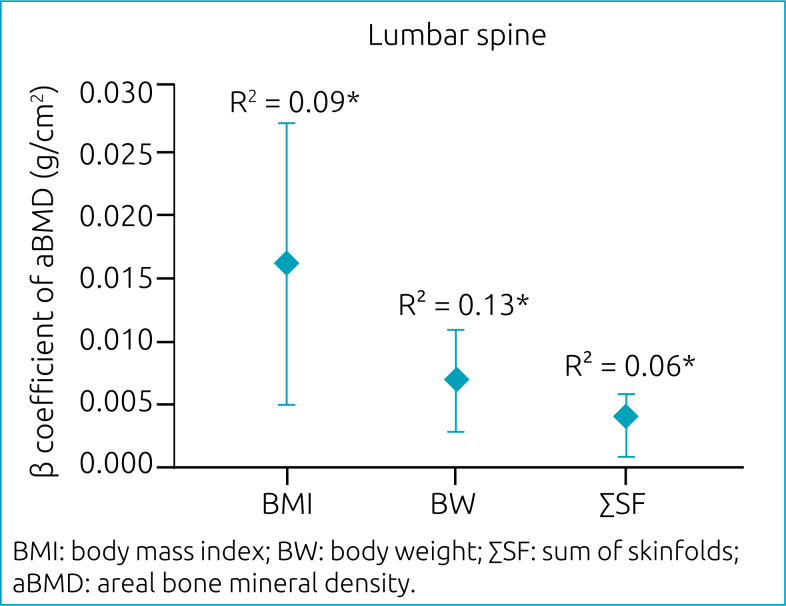
Association between body mass index, body weight, sum of skinfolds at childhood and areal bone mineral density at adulthood in males (n=69). Simple linear regression (p<0.01); *adjusted for chronological age.

For males, associations between childhood anthropometric indicators and aBMD in adulthood were positive, with the highest beta coefficients for BMI, followed by BW and ΣSF, with BMI explaining 9% (p<0.01), BW 13% (p<0.01), and ΣSF 6% (p<0.01) of aBMD only in the lumbar spine (Figure 2).

## DISCUSSION

The present study found that anthropometric indicators obtained in childhood are capable of predicting aBMD variations in early adulthood, especially in females, since anthropometric indicators from childhood showed positive association with all bone regions, while for males, aBMD was associated only with the lumbar spine region. Thus, identifying which factors in childhood and adolescence affect BM at subsequent ages can become an important strategy to delay and/or prevent the future appearance of bone tissue disorders.^27^

Among the investigated childhood anthropometric indicators, BMI was the variable with the greatest contribution to aBMD in adulthood, in both sexes. Bierhals et al.^14^ also found that BMI in late adolescence was the indicator that most contributed to BM in adulthood when compared with lean mass and fat mass. In the case of males, BMI in childhood was associated only with aBMD in the lumbar spine; a longitudinal study with a Chinese sample also found an association between BMI in adolescence and aBMD in the lumbar spine in adults, although it was also observed in other bone areas.^15^

In addition, it has recently been observed that fat-free mass seems to be an important mediator in the association between BMI and BMD for males;^15^ therefore, the importance of anthropometric and body composition changes during childhood and adolescence is highlighted, since gains in fat-free mass in males exceed those in females.^28^ Thus, it is believed that fat-free mass may impact males more strongly; however, this finding could not be confirmed in the present study.

For females, BMI was positively associated with all bone regions investigated, reflecting the contribution of BMI more notably for females. Contrary to these results, Foley et al.^17^ showed that BMI was not associated with BM; however, BM estimation was performed through ultrasonography of the calcaneal region; therefore, this comparison must be performed with caution.

Regarding the adiposity indicator (skinfolds), a positive association with aBMD was observed, but of lesser magnitude when compared to other anthropometric indicators, in both sexes. In agreement with the present result, Dong et al.^19^ identified positive associations between the subscapular skinfold in adolescence and BMD in adulthood. This can be explained by the stimuli to bone metabolism via cytokines, such as adipokines and steroid precursors, which in turn are associated with increased levels of circulating insulin and leptin.^29,30^ However, it is noteworthy that the relationship between fat mass and bone density seems to be ambiguous, since in obese children and adolescents, intra-abdominal adipose tissue was inversely proportional to total body BMD.^18^

Childhood and adolescence are important phases for BM accumulation, since the peak BM gain can occur up to 2 years after the height growth peak.^3^ Thus, the fact that this population is predominantly involved in daily activities that require body mass transport can contribute to bone health optimization, considering that bone modeling is sensitive to mechanical loads imposed by these activities.^7,8^

In brief, BMI was the main determinant of aBMD, and the mechanism involved in this process seems to be predominantly mechanical, considering that such a measure does not discriminate between lean and fat soft tissue or BM itself, and the effects of mechanical loads on BMD are due not only to the severity of BW (static), but also to dynamic loads through muscle contraction,^1^ even though lean mass is related to BM regardless of muscle fitness in children.^31^

It is noteworthy that, although the present study did not classify weight status, some studies have shown that overweight and obesity have a positive relationship with bone indicators.^22^ However, this fact should be analyzed with caution since young people classified as obese have lower BMC and BMD compared to their peers with normal weight and overweight,^9^ indicating the negative effect of excess adiposity on bone indicators via cellular mechanisms linked to body fat accumulation, which generates chronic low-grade inflammation and increases cytokine concentrations, negatively affecting bone health.^32^ Finally, the sex differences observed in associations between anthropometric measurements in childhood and BM in adulthood may partly reflect differences in body composition indicators between males and females in the contribution of bone development during growth.

Regarding limitations, the lack of information on physical activity, nutritional intake at baseline and follow-up, and DXA measurements at baseline stand out. Therefore, future studies are needed to investigate possible variations in childhood lean mass and fat mass in adulthood BM. Regarding strengths, we highlight the repeated measurements of the subjects obtained with an interval of 15 years, the use of anthropometric measurements that are easy to apply in children a nd adolescents, and the aBMD information obtained by DXA in adulthood in different anatomical regions. In addition, BMI has been widely used to classify nutritional status as it is easy to use in epidemiological and clinical surveys,^33,34^ and because it is one of the body size indicators in pediatric populations.

Anthropometric indicators obtained in childhood proved to be sensitive predictors of aBMD in adulthood, especially in females due to their association with all investigated bone areas, while for males, aBMD was associated only with the lumbar spine region. Among anthropometric indicators, BMI indicated a greater association with aBMD in both sexes.

## Data Availability

The database that originated the article is available with the corresponding author.
